# Introducing the Universal Periprosthetic Femur Fracture (UPFF) Classification: All Fractures Femur

**DOI:** 10.3390/jcm15051824

**Published:** 2026-02-27

**Authors:** Edward J. McPherson, Madhav Chowdhry

**Affiliations:** 1 Department of Orthopaedic Surgery, University of California Los Angeles, Los Angeles, CA 90404, USA; 2 Department of Continuing Education, Kellogg College, University of Oxford, Oxford OX1 2JA, UK

**Keywords:** periprosthetic, femur, fracture, classification, arthroplasty, trauma, hip, knee

## Abstract

Peri-Prosthetic Femur Fractures (PPFFs) are occurring with increasing frequency as the incidence of hip and knee arthroplasty is rising globally. PPFFs are presenting with more complex patterns commensurate with the increasing sophistication of implant technology and surgical technique. Moreover, with patients undergoing arthroplasty procedures at a younger mean age, it is not uncommon to see both ipsilateral implants (hip and knee) being affected by the fracture. Previous classifications have separated PPFFs into the hip or knee regions, and most do not include fracture patterns about revision-style implants. Prior schemes are antiquated and are not applicable to all current fracture patterns. We present a codified anthropometric, rule-based staging system that incorporates PPFFs across the entire femur. This system is designed to standardize communication and capture variables relevant to treatment planning and future research analysis. Formal reliability and clinical validation studies are ongoing.

## 1. Introduction

Total Hip Arthroplasty (THA) and Total Knee Arthroplasty (TKA) procedures continue to increase globally [1,2,3]. Expectantly, revision procedures are increasing, as second- and third-generation implants require revision. Part of this increase relates to Peri-Prosthetic Femur Fracture (PPFF), with multiple arthroplasty registries reporting increasing incidence [4,5]. Moreover, fractures are presenting with more complex patterns. This is related to implant designs, stem length, surgical technique, regional femoral bone density changes, and evolving fixation methods, including hybrid implant fixation techniques. In addition, new fracture patterns are occurring with the adoption of precision alignment systems that require the placement of adjacent bony registration pins [6,7,8]. The above factors, when combined, create stress risers at various positions within the femur that are patient-unique. Forces imparted to the femur, whether from normal activities of daily living (ADL) or excess stress from falls, will focus energy at these stress riser points creating unique fracture patterns. Not infrequently, patients have concomitant ipsilateral THA and TKA implants of differing ages, creating distortions of femoral bone bending and torsion [9,10,11]. In addition, fractures are occurring in the presence of revision medullary stems of various lengths, and now, it is not uncommon to see fractures in between medullary TKA and THA stems, known as inter-prosthetic fractures [12,13,14].

PPFFs generally occur early or late within the lifecycle of implants. Early fractures occur within the first 10–12 weeks, driven by surgical technique, implant design, and rehabilitation protocols [15,16,17,18]. These fractures occur from cortical tube disruptions and/or stress risers created during prosthetic insertion. These fractures occur either acutely due to direct force trauma or sub-acutely through repetitive loading, leading to overt fracture, prosthetic loosening, or both [16,19]. Conversely, late-onset PPFFs occur beyond this period at points of modulus mismatch, where localized bone overload, triggered by trauma or physiological stress on weakened bone, initiates the fracture. This mismatch is common at the stem tip, occurring with large-diameter uncemented stems or at the junction of cemented stems and a thin distal cortical tube. Additionally, bone compromise from infection, mechanical loosening, or particulate debris reactions can further exacerbate these stress risers [18,19,20].

Subspecialty training affects treatment choice. PPFF management is within the purview of trauma and arthroplasty surgeons, with both groups differing in treatment philosophy. Trauma specialists typically focus on fracture fixation using plates and medullary nails, with bone quality being important in treatment selection. Arthroplasty surgeons, in contrast, consider implant longevity, bone debris reactions, and future-needed joint surgery, often opting to replace the implant and extend the prosthetic construct to secure the fracture. A new classification system must embrace the key priorities of both subspecialties.

Many classification schemes for PPFFs have been reported [21,22,23,24,25,26,27,28,29,30,31,32,33,34,35,36,37]. PPFF schemes are typically separated into hip and knee regions and do not specifically address revision implants. For the hip, the commonly used Vancouver hip scheme is antiquated due to new fracture patterns occurring in differing stem designs, creating “classification conflict” [14,30,38]. Knee fracture schemes are numerous but fail to consider the downstream implications of the hip and inter-prosthetic regions [33,39,40,41]. Trauma-developed classifications fail to consider implant factors, including bearing wear, bone debris reactions, kinematic joint stability, and the presence of associated hardware [13,30,42]. Acknowledging the inherent specialty priorities, we introduce a classification scheme that encompasses all PPFFs, hip to knee, which we name Universal Periprosthetic Femur Fracture (UPFF) classification. Our aim with this classification is to provide a codified scheme acceptable to all, with the goal of providing salient guidance for PPFF treatment. This manuscript presents the classification in detail, describing the steps in the classification with illustrations, and then providing radiographic case examples.

## 2. Universal Periprosthetic Femur Fracture (UPFF) Classification

Design goals for the UPFF classification include:

1. Descriptive communication:
  - The framework is designed to provide a concise alphanumeric description that supports an intuitive mental visualization of the fracture and implants, avoiding the need for rote memorization.
  - The system provides descriptive rules without the need for calculations, allowing efficiency in communication.
2. Cross-discipline usability:
  - The framework is designed for use by all orthopedists, including generalists and trauma and arthroplasty surgeons, reducing specialty-dependent interpretation.
3. Clinical actionability:
  - The included elements were specifically selected to provide information relevant to surgical planning and execution.
4. Operational simplicity:
  - The descriptive system is designed to allow physicians and surgeons of various training to communicate effectively amongst their peers, utilizing defined steps.
5. Pan-femoral inclusivity:
  - Its structure is designed to permit the incorporation of all periprosthetic femur fractures, hip to knee, and to incorporate all future fracture patterns without redefining its core principles.
  - Fracture patterns yet unknown can be incorporated into the scheme, possibly with the addition of a new modifier. This flexibility will prevent the classification scheme from becoming obsolete.
6. Fidelity in research stratification:
  - Classifying PPFF by location, bone quality, implant fixation and modifiers will allow each fracture type to be included as individual MeSH (Medical Subject Heading) keywords. For academic study, this scheme may reduce research heterogeneity and allow critical analysis of outcome measures based upon specified fracture patterns.
7. Future automation/Artificial Intelligence (AI) applicability:
  - Because UPFF is anthropometrically defined and rule-based, it may be suitable for future development of AI-based classification tools. This will require a large dataset development and validation.
  - An AI-trained model may assist in immediate classification, aiding all healthcare personnel from trainees to senior staff.
  - Stratification of PPFF by AI may assist in research analysis, potentially enhancing evidence-based, recommended treatment for specified fracture patterns.

## 3. Basic Rules of UPFF

### 3.1. Order and Sequencing

- Letters and numbers describing a fracture start from the top (superior) of the femur and proceed towards the knee.
- Classification is based upon Anterior–Posterior (AP) radiographic views of the entire femur. Orthogonal views and Computer Tomography (CT) are supplementary, confirming fracture extent, implant stability, and additional bone findings, ordered as needed.
- By convention, a fracture is periprosthetic when it originates or ends within the same fracture zone of an arthroplasty implant.

#### 3.1.1. Fracture Location

**Figure 1:**
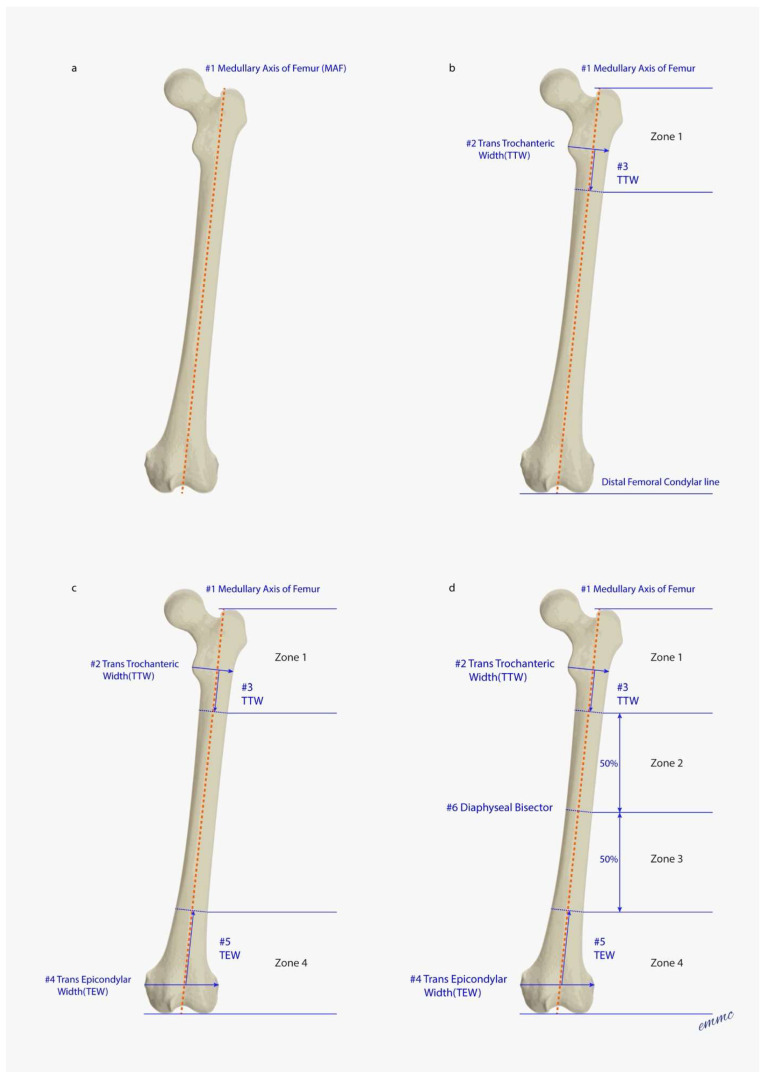
(**a**–**d**) These 4 figures define in sequence the fracture zones illustrating the process step by step to arrive at the final zone diagram in Figure 2. (**a**) Draw Medullary Axis of Femur (MAF) (Step #1). (**b**) Define Zone 1. First, draw the distal femoral condylar line, which extends between the tangents of the medial and lateral femoral condyles. Then, draw a line at the Greater Trochanteric tip that is parallel to the distal femoral condylar line. Next, draw the Trans-Trochanteric Width (TTW) starting at the medial lesser trochanteric apex, extending to the lateral outer femoral cortex. The line is perpendicular to MAF (Step #2). Take the TTW and rotate it to be parallel and next to MAF (Step #3). The end of TTW defines the lower limit for Zone 1. (**c**) Define Zone 4. First, draw the Trans-Epicondylar Width (TEW) starting at the medial epicondylar apex, extending to the lateral epicondylar apex (Step #4). Second, take the TEW and rotate it to be parallel and next to MAF (Step #5). The end of the TEW defines the upper limit for Zone 4. Zone 4 extends distally to the distal femoral condylar line. (**d**) Define Zones 2 and 3. First, identify the MAF between the Zone 1 bottom limit and the Zone 4 top limit and split into two equal segments (Step #6). This bisector line creates Zone 2 (above) and Zone 3 (below).

The femur is divided anthropometrically into 4 fracture zones, which are defined below (Figure 1a–d). Zone 1 and Zone 4 are defined first in order to determine Zones 2 and 3:

- **Zone 1:** Starts from the proximal tip of the greater trochanter, extending one trans-trochanteric width (TTW) (of the lesser trochanter) distal to the TTW along the femoral medullary axis. The TTW is defined as the width measured at the medial lesser trochanteric apex to the outer lateral femoral cortex.
  Rationale: Femur lengths vary widely. The TTW provides a proportional length reference to define the inferior extent of Zone 1 [43,44,45].
- **Zone 4:** Starts from a line joining the distal femoral condyles, extending one trans-epicondylar width (TEW) proximal to the TEW along the femoral medullary axis (i.e., extending proximal to the meta-diaphyseal knee flare). The TEW is defined as the line joining the medial and lateral epicondyles.
  Rationale: Femur lengths vary widely. The TEW provides a proportional length reference to define the superior extent of Zone 4 [43,44,45].
- **Zone 2:** The femoral diaphysis is divided into two equal sections between Zone 1 and Zone 4. Zone 2 begins from the inferior extent of Zone 1 to the top of Zone 3.
- **Zone 3:** The femoral diaphysis is divided into two equal sections between Zone 1 and Zone 4. Zone 3 begins from the inferior extent of Zone 2 to the top of Zone 4.

**Figure 2:**
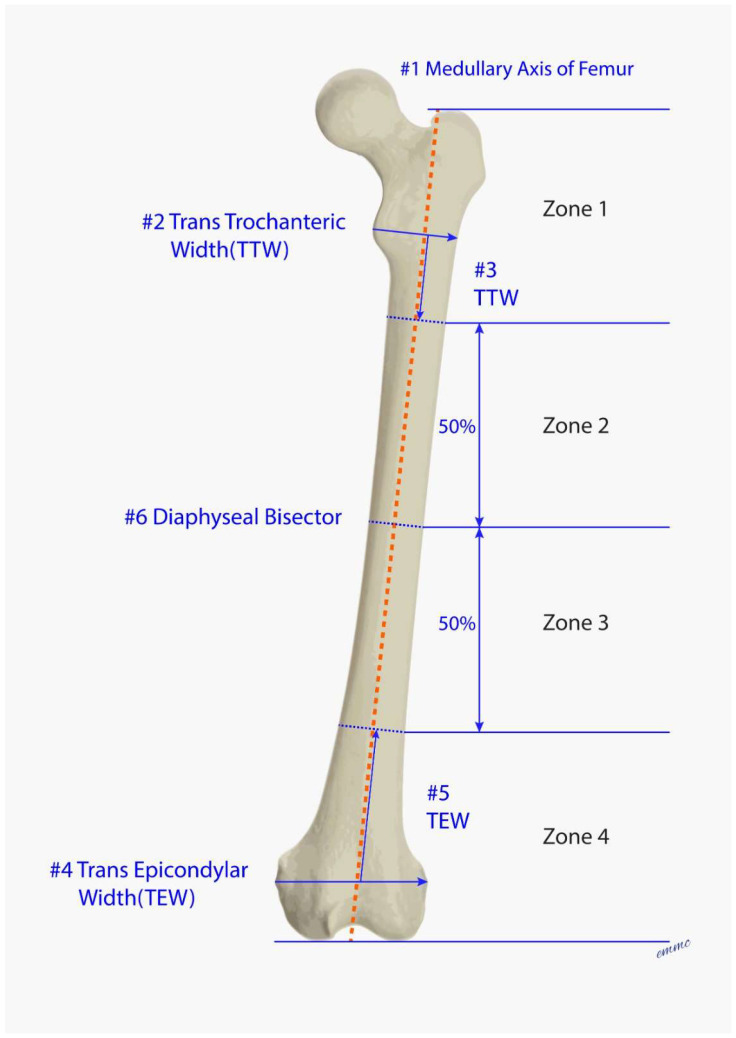
This combined figure displays the 4 fracture zones and all steps in defining the zones, which are numbered #1 through #6. Knowing the rules above, the shortcut method is to take the TTW and turn it sideways to define the bottom of Zone 1. Take the TEW and turn it sideways to define the top of Zone 4, and then, split the remaining femur into 2 equal sections.

The sequence for defining the 4 fracture zones is outlined in Figure 1 and Figure 2.

The fracture is defined numerically by the number of zones involved. The fracture by convention includes all fractured zones, starting from the most proximal zone to the most distal zone of involvement. There are 10 possible fracture descriptors, which are intuitive. All possible fracture configurations are described in Table 1.

#### 3.1.2. Bone Quality

Bone quality is of high priority to determine the adequacy of screw fixation [46,47,48,49]. Bone quality is assessed by a simple determination of cortical thickness at the fracture and is divided into three bone descriptors.

**Figure 3:**
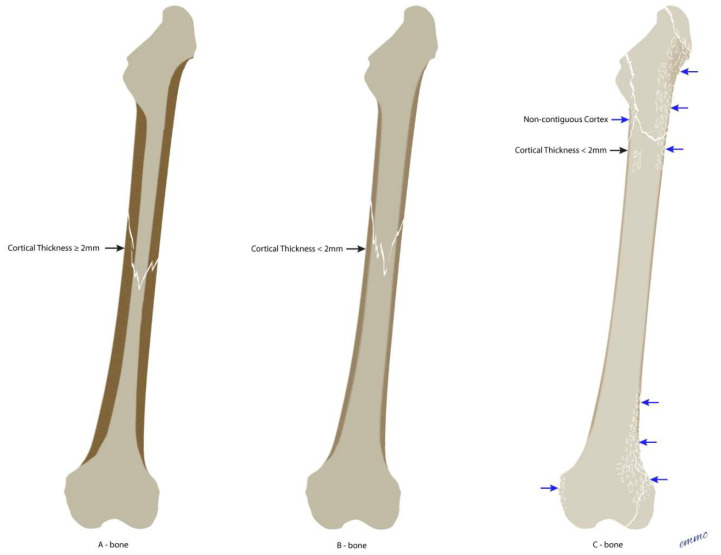
(**A**–**C**) Diagrammatic illustrations of bone quality descriptors. (**A**) Diagram of A (Adequate) bone. Bone is adequate for screws with a cortex thickness ≥ 2 mm in fracture zone(s). (**B**) Diagram of B (Bad) bone. Bone is likely inadequate for screws with a cortex thickness < 2 mm in fracture zone(s). (**C**) Diagram of C (Chitty) bone. Bone is non-contiguous and riddled (like individual chits of paper) and will not hold screws.

By convention, bone thickness is described by the thinnest cortical bone within the fracture zone(s). Table 2 describes the three bone types, and Figure 3 illustrates the three bone quality descriptors.

#### 3.1.3. Implant Descriptors

Describing implant status determines the need for implant revision, which shapes preoperative planning. A three-letter acronym is used. The implant descriptors are the following:

- HL — Hip Loose
- HS — Hip Stable
- KL — Knee Loose
- KS — Knee Stable
-   C — Cemented
-   U — Uncemented

The three-letter implant descriptor is stated in this order: hip/knee, stable/loose, and cemented/uncemented. The implant descriptor is stated after bone quality. All possible implant descriptors are presented in Table 3.

The parameters defining a loose cemented and uncemented stem are complex and will be judged by the treating orthopedic team and later verified intra-operatively. In an acute fracture, a cemented stem (hip or knee) by convention is loose when the fracture disrupts >1/3 of the bone-cement mantle [36,37,40,50,51,52,53].

#### 3.1.4. Modifiers

Modifiers describe additional fracture characteristics that require an actionable change or adaptation in the treatment plan. Modifiers are two-letter descriptors. Modifiers follow the implant descriptor. All relevant modifiers are to be listed when present.

- GT — Greater trochanter separation—state separation in (cm) with brackets
- LT — Lesser trochanter separation—state separation in (cm) with brackets
- ST — Stem present (at knee)
- IS  — Inter-stem fracture—state distance in between stems in (cm) with brackets
- SG — Segmental fracture (more than 2 main segments)
- BS — Broken Stem
- BP — Broken Plate/Nail
- CB — Closed box knee prosthesis (if recognizable)
- IF  — Infection present
- MT— Metastatic tumor
- OF — Open fracture

### 3.2. Staging Steps

*Step #1—State Fracture Location*
  - Fracture location is described using the 4 defined femoral zones
  - One zone fracture—state the femoral zone of involvement
  - Multi-zone fracture—state first the most proximal femoral zone of fracture, followed by the most distal femoral zone of fracture.
  - Examples: 2, 4, 13, 24, etc.
*Step #2—State Bone Quality*
  - Cortical bone quality descriptor will follow the fracture zone, and by convention, there will be no space.
  - Examples: 1B,12B, 34A, 23C, etc.
*Step #3—State Implant Descriptor*
  - Implant descriptors follow bone quality. By convention, the implant descriptor will be preceded by a hyphen.
  - By convention, in cases of ipsilateral femoral implants, state the descriptor for both the hip and knee, starting with the hip descriptor first. Place a comma between the hip and knee descriptors.
  - Examples: 12B-HLU, 23B-HLC, 4B-KSC, 13B-HLU, KSC, etc.
*Step #4—State Modifiers*
  - Modifiers follow implant descriptors, and by convention will be preceded by a hyphen.
  - If there are no modifiers, this section is left blank.
  - State all applicable modifiers. If multiple modifiers are used, place a comma between each modifier.
  - When discussing by telephone, the modifiers may be described, rather than using their acronym, for ease of communication.
  - Examples: 23A-HLC-IF, 1B-HLU-GT (3 cm), 4A-KSC-CB, 34A-KLC-ST, etc.

### 3.3. Examples of UPFF Classification with Translation

Note: the oratory translation can vary (i.e., presentation style), but all descriptors must be included.

1. 1C-HSU-GT (2.5 cm)
  - Fracture of the proximal hip, with chitty bone (riddled bone) (e.g., massive osteolysis), with a stable uncemented hip implant, with proximal displacement of the Greater Trochanter by 2.5 cm.
2. 2B-HSC
  - Fracture of the meta-diaphyseal hip, with bad bone, with a stable cemented hip implant.
3. 34B-KSC-SG
  - Fracture of the meta-diaphyseal knee, with bad bone, with a stable cemented knee implant, with a segmental fracture above.
4. 34B-KLC-ST
  - Fracture of the meta-diaphyseal knee, with bad bone, with a loose cemented knee implant, with a stem.
5. 23A-HSU, KSC-ST, IS (5 cm); this could also be written as 23A-HSU, KSC-IS-5 cm.
  - Fracture of the diaphysis, with adequate bone, with a stable uncemented hip implant, with a stable cemented knee implant with a stem, with an inter-stem fracture with 5 cm stem tip separation.
  - Alternative description: Inter-stem fracture between fixed uncemented THA and fixed cemented TKA with a stem, adequate bone, and stem separation of 5 cm.
6. 13A-HLU-SG
  - Fracture of the proximal 3/4 femur, with adequate bone, with a loose uncemented hip implant, with a segmental fracture.
7. 4B-KSC
  - Fracture of the distal knee, with bad bone, with a stable cemented knee implant. Note: since the CB modifier is not included, an intramedullary stem is permissible.
8. 2A-HLU-IF
  - Fracture of the meta-diaphyseal hip, with adequate bone, with a loose uncemented hip implant, with infection.
9. 24A-KLC-ST, SG
  - Fracture of the distal 3/4 femur, with adequate bone, with a loose cemented knee implant with stem, with a segmental fracture.
10. 23B-HSU-MT
  - Fracture of the diaphysis, with bad bone, with a stable uncemented hip implant, with metastatic tumor (e.g., multiple myeloma).

### 3.4. Radiographic Cases of UPFF Classification (Table 4)

This section illustrates the entire process defining a PPFF employing the UPFF classification. The radiographs shown are classified according to the above rules. For manuscript brevity, only relevant radiographs are displayed, and the rest of the femur is assumed to be not involved (Table 4). 

**Table 4:** Radiographic examples with UPFF classification with brief translation. Note: For manuscript clarity, only salient radiographs are shown to illustrate the alphanumeric fracture classification.

UPFF Classification	Description	Radiograph 1	Radiograph 2
**1B-HSU-GT (2 cm)**	Fracture zone 1, bad bone (lateral view), hip stable uncemented, greater troch w/2 cm displacement	** 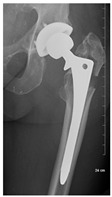 **	** 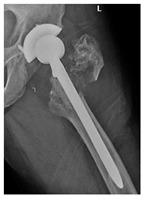 **
**1B-HSU-GT (1.0 cm), LT (0.5 cm)**	Fracture zone 1, bad bone, hip stable uncemented, greater troch w/1 cm & lesser troch w/0.5 cm displacement	** 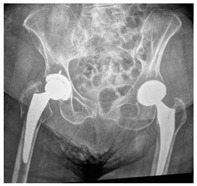 **	** 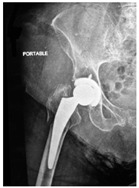 **
**2A-HSC-SG**	Fracture zone 2, adequate bone, hip stable cemented, segmental	** 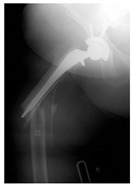 **	
**2C-HLC**	Fracture zone 2, chitty bone, hip loose cemented (>1/3 of cement mantle disrupted)	** 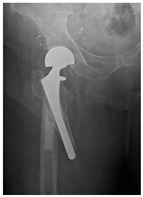 **	** 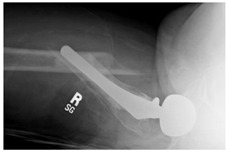 **
**12A-HLC**	Fracture zone 1 through 2, adequate bone, hip loose cemented (>1/3 of cement mantle disrupted)	** 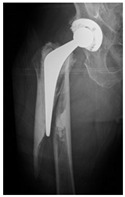 **	
**12A-HLU**	Fracture zone 1 through 2, adequate bone, hip loose, uncemented	** 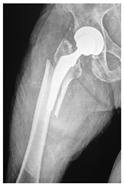 **	** 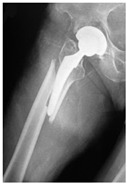 **
**12A-HLU-SG**	Fracture zone 1 through 2, adequate bone, hip loose uncemented, segmental	** 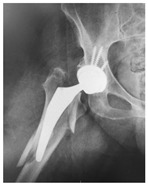 **	** 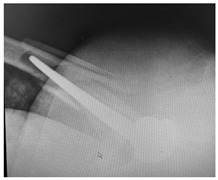 **
**12A-HSU**	Fracture zone 1 through 2, adequate bone, hip stable, uncemented (note: image on the right is a 3D CT scan)	** 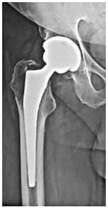 **	** 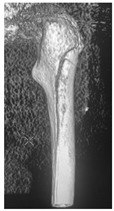 **
**13B-HLC**	Fracture zone 1 through 3, bad bone, hip loose cemented	** 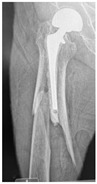 **	** 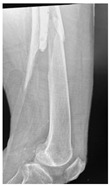 **
**2A-HSU-BP**	Fracture zone 2, adequate bone, hip stable uncemented, broken plate	** 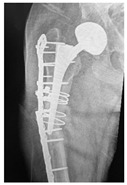 **	** 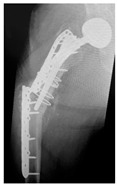 **
**2B-HSU-BS**	Fracture zone 2, bad bone, hip stable uncemented, broken stem	** 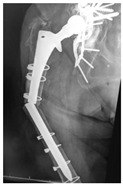 **	
**3A-HSU, KSC-ST, IS (6.5cm), BP**	Fracture zone 3, adequate bone, hip stable uncemented, knee stable cemented w/stem, inter-stem w/6.5 cm separation, broken plate	** 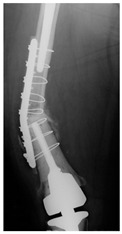 **	** 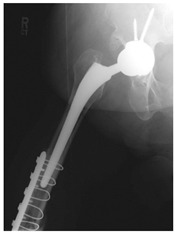 **
**14A-HLU, KSC-SG**	Fracture zone 1 through 4, adequate bone, hip loose uncemented, knee stable cemented, segmental	** 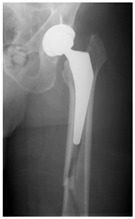 **	** 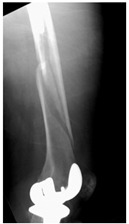 **
**2C-KLC-ST**	Fracture zone 2, chitty bone (lateral cortex), knee loose cemented, knee stem	** 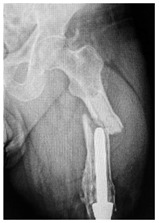 **	** 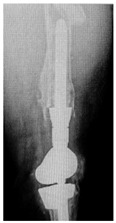 **
**24A-KLC-ST**	Fracture zone 2 through 4, adequate bone, knee loose cemented (>1/3 of cement mantle disrupted), knee stem	** 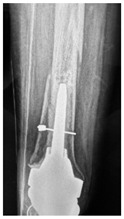 **	** 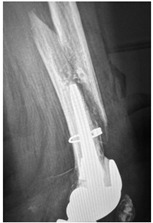 **
**34A-KLC-ST**	Fracture zone 3 through 4, adequate bone, knee loose cemented (>1/3 of cement mantle disrupted), knee stem	** 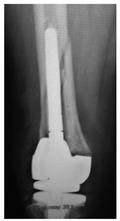 **	** 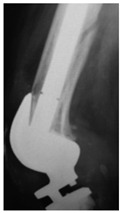 **
**34A-KLC-ST**	Fracture zone 3 through 4, adequate bone, knee loose cemented, knee stem	** 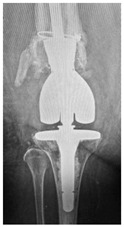 **	** 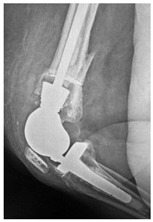 **
**34A-KLC**	Fracture zone 3 through 4, adequate bone, knee loose cemented (>1/3 of cement mantle disrupted) knee stem. Note: knee is endofusion	** 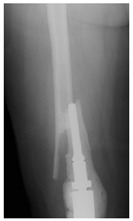 **	
**34A-HSU, KSC-SG**	Fracture zone 3 through 4, adequate bone, hip stable uncemented, knee stable cemented, segmental	** 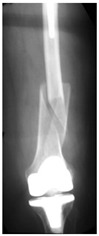 **	** 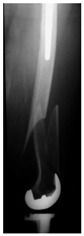 **
**4B-KSC**	Fracture zone 4, bad bone, knee stable cemented	** 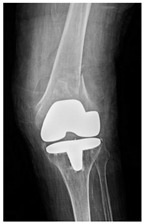 **	** 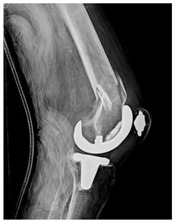 **
**4A-KSC**	Fracture zone 4, adequate bone, knee stable cemented	** 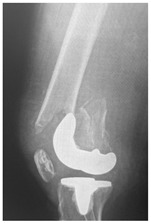 **	
**4C-KLC**	Fracture zone 4, chitty bone (at fracture site), knee loose, cemented	** 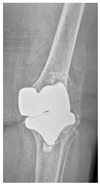 **	** 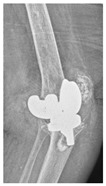 **

## 4. Discussion

The treatment of PPFF is evolving in an effort to keep pace with its growing incidence and the new fracture variants seen with more sophisticated arthroplasty reconstructions. PPFF classifications introduced decades prior may not accommodate all currently observed fracture patterns without “stretching” antiquated definitions, which can create classification conflict and reduce data fidelity [21,22,23,24,25,26,27,28,29,30,31,32,33,34,35,36,37,39]. For example, a pseudo AL fracture in the Vancouver classification system (VCS) with a loose stem conflicts with the only loose stem descriptors in VCS, which are stem tip fractures B2 and B3 [54]. Fracture patterns that do not truly fit a category may distort outcomes and bias advanced analyses, including systematic reviews and meta/network analysis. More importantly, we currently lack a classification scheme incorporating all PPFFs that includes salient factors that are actionable in guiding treatment. Lastly, there is no widely accepted scheme adopted by both trauma and arthroplasty specialists. This classification bridges these limitations [36].

Table 5 compares the current UPFF to prior commonly used PPFF schemes published since 1995. UPFF differs by separating fracture zones based on anthropometric dimensions, rather than prosthetic dimensions, providing uniformity. It also includes descriptors of bone quality useful for fixation and considers variables involving prosthetic implants requiring actionable treatment. Whether these differences translate into improved communication, clinical utility, and data fidelity will require formal validation. UPFF is a conceptual, rule-based descriptive framework, and clinical outcomes have not yet been established. Certain elements, particularly implant stability and cement mantle disruption, are partly subjective and dependent on imaging quality and intraoperative findings. However, we predict that UPFF will offer clearer guidance for clinical decision-making and provide a more standardized, evidence-generating framework for research compared to recently published schemes.

In the last decade, we have seen a surging number of classification and staging schemes encompassing numerous areas within orthopedics. For orthopedists at any level of training, it is challenging to memorize all schemes. Our philosophy in developing the UPFF classification was to make it practical, with explicit recognition that adoption depends on demonstrated simplicity, reliability, and usability. Accordingly, UPFF is proposed as a descriptive framework designed to be intuitive and easy to apply; its uptake and impact will require formal validation and user testing. This is our goal.

The UPFF classification is anthropometrically designed, based on standardized bone fracture zones, as opposed to being “prosthetic-centric,” which creates significant variability. The design scheme is flexible enough to accommodate evolving prosthetic technologies. While we recognize that new classifications often face initial resistance, the need for a universal PPFF system is clear. Our current focus is on establishing inter- and intra-observer validation at an independent academic center, with plans for multicenter trials to establish clinical outcome reliability. Further, we plan the future validation of this scheme across several trained AI models. We believe this universal system will significantly benefit the orthopedic community by providing consistent stratification and allowing improved outcome analysis.

## 5. Conclusions

We present a classification for PPFF that incorporates fracture location across the entire femur while encoding bone quality, implant condition, and relevant modifiers using an intuitive alphanumeric descriptor. The framework is designed to be extensible, allowing the incorporation of emerging fracture patterns without redefining core zones. By employing the UPFF acronyms in research articles, we anticipate reduced heterogeneity and better clarity of the analyzed outcome measures. We are currently proceeding with inter- and intra-observer validity testing. Subsequent studies will evaluate clinical validity, including associations with treatment choice and outcomes, and whether the use of UPFF reduces heterogeneity in research reporting. Because UPFF is anthropometrically defined and rule-based, it may be suitable for future development of AI-based classification tools. This will require a large dataset development and validation. The goal is to reach consensus treatment by surgeons working in this difficult field.

## Figures and Tables

**Table 1:** Fracture descriptors.

Fracture Description	Zones of Fracture Involvement
1	Zone 1 only
12	Zone 1 and 2
13	Zone 1 through 3
14	Zone 1 through 4
2	Zone 2 only
23	Zone 2 and 3
24	Zone 2 through 4
3	Zone 3 only
34	Zone 3 and 4
4	Zone 4 only

**Table 2:** Bone quality descriptors.

Bone Type	Definition	Implication
**A**–Bone (**A**dequate)	Cortical thickness ≥ 2.0 mm	Adequate bone for screws
**B**–Bone (**B**ad bone)	Cortical thickness < 2.0 mm	Screws may be possible
**C**–Bone (**C**hitty * bone)	Bone is riddled	Inadequate bone for screws

Bolded letters (A, B, C) are used as a mnemonic corresponding to adequate, bad and chitty. * Chitty—cortical bone is riddled (like chits of paper) from comminution, osteolysis, infection, or metastatic neoplasia.

**Table 3:** Three letter acronyms for implant descriptors.

Implant Acronym	Acronym Description
HLC	Hip Implant Loose — Cemented
HLU	Hip Implant Loose — Uncemented
HSC	Hip Implant Stable — Cemented
HSU	Hip Implant Stable — Uncemented
KLC	Knee Implant Loose — Cemented
KLU	Knee Implant Loose — Uncemented
KSC	Knee Implant Stable — Cemented
KSU	Knee Implant Stable — Uncemented

**Table 5:** Comparison of UPFF to published recent periprosthetic femur fracture classifications used in the last 20 years.

	UPFF	VCS ^b^	UCPF/OTA ^a,b^	Lewis/ Rorabeck	Su et al.	Baba et al.
Year introduced	2026	1995 [23]	2014 [21]	1999 [33]	2004 [39]	2015 [36]
Rating Regions	Hip to Knee	Hip & Femur	Hip & Femur	Knee only	Knee & Femur	Prosthetic Stability Hip &/or Knee only
Anthropometrically defines femur fracture zones	Yes	No	No	No	No	No
Bone quality rating	Quantitative + Descriptive	Qualitative	Qualitative	No	No	No
Classifies entire femur	Yes	No	Yes-separate schemes hip & knee	No	No	No
Simultaneous hip & knee implant description	Yes	No	No	No	No	No
Classifies multi-zone fractures	Yes	No	No	No	No	No
Classifies inter-stem fractures	Yes	No	Yes	No	No	No
Implant descriptors: (e.g., knee stem, closed box)	Yes	No	No	No	No	Yes
Implant fixation description: cemented vs. uncemented	Yes	No	No	Yes	No	Yes
Broken hardware descriptor (stem, implant or plate)	Yes	No	No	No	No	No
Expandable for new conditions	Yes	No	No	No	No	Yes
Intuitive	Yes	Yes	No	Yes	Yes	No

^a^ UCPF (Universal Classification for Periprosthetic Fractures) is the default classification for AO/OTA (Arbeitsgemeinschaft für Osteosynthesefragen/Orthopedic Trauma Association) for periprosthetic femur fractures. ^b^ Includes sub-classifications of UCPF and VCS (Vancouver Classification System).

## Data Availability

No new data were created or analyzed in this study.
